# Li–Fraumeni Syndrome: Narrative Review Through a Case Report with Ten Years of Primary Tumor Remission Associated with *Sechium* H387 07 Supplementation

**DOI:** 10.3390/ijms252111477

**Published:** 2024-10-25

**Authors:** Angel Iván Amador-Gómez, Itzen Aguiñiga-Sánchez, Víctor Manuel Mendoza-Núñez, Jorge Cadena-Iñiguez, Ernesto Romero-López, Edelmiro Santiago-Osorio

**Affiliations:** 1 Hematopoiesis and Leukemia Laboratory, Research Unit on Cell Differentiation and Cancer, Faculty of High Studies Zaragoza, National Autonomous University of Mexico, Mexico City 09230, Mexico; angelivan.98@icloud.com (A.I.A.-G.); liberitzen@comunidad.unam.mx (I.A.-S.); dr.ernestoromerolopez@gmail.com (E.R.-L.); 2 Department of Biomedical Sciences, School of Medicine, Faculty of Higher Studies Zaragoza, National Autonomous University of Mexico, Mexico City 09230, Mexico; 3 Research Unit on Gerontology, FES Zaragoza, National Autonomous University of Mexico, Mexico City 09230, Mexico; mendovic@unam.mx; 4 Postgraduate College, Campus San Luis Potosí, Salinas de Hidalgo, San Luis Potosí 78622, Mexico; jocadena@colpos.mx

**Keywords:** Li–Fraumeni syndrome, precancerous niche, tumor microenvironment, cancer chemoprophylaxis, *TP53*, polyphenols, *Sechium edule*

## Abstract

There are hereditary mutations that predispose individuals to cancer development, such as pathogenic variants in the germ line of the tumor protein 53 (*TP53*) suppressor gene. This leads to a rare condition known as Li–Fraumeni syndrome (LFS), characterized by a high risk of developing multiple cancers throughout life by the precancerous niche that promotes the tumor microenvironment. LFS presents a significant challenge due to its limited therapeutic and chemoprophylactic options. Recently, protocols involving metformin as a prophylactic medication have been developed to target precancerous niches. However, this approach is still in the clinical phase, and no established therapeutic regimen is available. Therefore, new alternatives are needed to impact this disease effectively. Novel studies suggest that *Sechium* extract, rich in polyphenols, exhibits chemoprophylactic, antineoplastic, anti-inflammatory, and antioxidant activities, all involved in the tumor microenvironment of LFS. However, the specific role of *Sechium* extract in preventing recurrent neoplastic development in LFS remains unclear. We conducted this research through a case report of an LFS-diagnosed patient who has experienced multiple malignancies and cutaneous neoformations. This patient received a chemoprophylactic supplementation based on *Sechium* H387 07 extract over 11 years without reporting new primary malignancy events or recurrences, as evidenced by laboratory and positron emission tomography/computed tomography (PET/CT) studies. An extensive literature review on the disease, precancerous niche, tumor microenvironment, and potential mechanisms of *Sechium* H387 07 extract components was conducted to explain cancer absence in LFS. This review promotes the research and use of polyphenols as powerful chemoprophylactic agents to prevent and treat proliferative diseases like LFS.

## 1. Introduction

### 1.1. LFS and TP53

Li–Fraumeni syndrome (LFS; OMIM 151623) was first described by Frederick P. Li and Joseph F. Fraumeni in 1969. They observed four families with early-onset soft tissue sarcomas, breast cancer, and other neoplasms [1]. However, it was not until 1990 that germline pathogenic variants in the *TP53* gene (*mTP53*) were identified as the underlying cause of LFS [2]. The *TP53* tumor suppressor gene is located on the *17p* chromosome [3]. It encodes p53, often called the “guardian of the genome”. p53 plays a crucial role in DNA repairment, cell cycle regulation, apoptosis, and metabolic changes, which are essential for cancer prevention [4,5,6].

LFS is a rare autosomal dominant disorder with a penetrance ≥75% in males and nearly 100% in females [7]. The American College of Medical Genetics and Genomics (ACMG) consider the mutations in TP53 “likely pathogenic” and “pathogenic” as those of clinical significance [2,6].

### 1.2. Epidemiology of LFS

There is a record of more than 3034 individuals from 1282 families with significant pathogenic variants in *TP53* [2]. Approximately 500 families with LFS have been reported [8]. As age advances, the accumulative risk of malignancy increases, reaching 50% by age 40 and 100% by age 70. The risk of a second malignancy within ten years is >50% [7,9,10,11]. Multiple alterations can predispose to secondary cancer development, even oncological treatment as a consequence of primary tumors [12], impacting the quality of life and giving a prognosis associated with site and age of malignancy presentation [2,3,13].

Despite being a rare condition, there is significant worldwide variation in its prevalence. Brazil, for instance, is estimated to have around 300,000 carriers with some pathogenic variant. Interestingly, these carriers exhibit a lower penetrance (50–60%) and a reduced lifetime risk of developing cancer compared to the typical LFS phenotype [11,14]

The low prevalence hinders studies [8], and no evidence supports effective chemoprophylaxis, with actions focused on reducing exposure to carcinogens [7].

### 1.3. Diagnostic Approach and Prevention

The diagnostic suspicion for LFS is guided by the National Comprehensive Cancer Network (NCCN) criteria:

- Classic LFS criteria:
  - A member of a kindred with a known *TP53* pathogenic or likely pathogenic variant.
  - A combination of an individual diagnosed at 45 years or younger with a sarcoma first-degree relative diagnosed with cancer at 45 years or younger.
  - And an additional first- or second-degree relative in the same lineage with cancer diagnosed at younger than 45 years or a sarcoma diagnosed at any age.
- Chompret criteria:
  - Multiple primary tumors of at least 2 “core” LFS tumor types (sarcoma, breast cancer, adrenocortical carcinoma, brain tumors) diagnosed at <36 years or patients with adrenocortical carcinoma diagnosed at any age, regardless of family history.

Considering both sets of criteria, detecting *TP53* pathogenic variants has a sensitivity of up to 95%, requiring a genetic test for definitive confirmation. It is advised that individuals from families harboring pathogenic *TP53* variants seek genetic testing, even if the cancer has not been diagnosed [15].

Current guidelines for early cancer screening include physical examinations, laboratory, and imaging studies starting at 18 years for LFS-diagnosed patients or even from birth for descendants of families with *mTP53* [2,6,16,17]. In addition to close oncological follow-up, bilateral prophylactic mastectomy is suggested due to the high risk of breast cancer [14]. Patients already diagnosed with cancer should be limited to the use of genotoxic radiotherapy and chemotherapy, which are crucial to prevent secondary malignancies [6,12,18].

### 1.4. The Cancer Diversity Within LFS

LFS predisposes individuals to a wide range of malignancies associated with age:

- 0 to 15 years: adrenocortical carcinoma, rhabdomyosarcoma, choroid plexus carcinoma, and medulloblastoma.
- 16 to 50 years: osteosarcoma, leukemia, gliomas, breast cancer, lung cancer, and gastrointestinal cancer.
- ≥51 years: prostate cancer and pancreatic cancer [2,3].

While typical phenotype involves breast cancer and soft tissue tumors, regional variations exist. For instance, in Brazil, there is a higher incidence of adrenocortical, thyroid, and breast tumors, while in Japan, stomach, colorectal, lung, and liver cancers are more prevalent [11,19]. Additionally, LFS is not only determined by genetics. Identical *mTP53* twins have demonstrated different disease phenotypes, suggesting the role of environmental factors in their development [20].

### 1.5. The Precancerous Niche in LFS

The pathogenesis underlying malignancy in LFS is associated with *TP53* function. Researchers have proposed the existence of a precancerous niche, as suggested by Kaplan and Peinado [21,22]. This niche is characterized by a tumor microenvironment that promotes malignant proliferation. Critical processes within this context include chronic inflammation, uncontrolled oxidative stress, pro-angiogenic signaling, immune dysregulation, metabolic reprogramming, and faulty apoptosis pathways. In the presence of insults, these factors can predispose individuals to various cancers [13].

### 1.6. Intervening on Precancerous Niche

**Figure 1:**
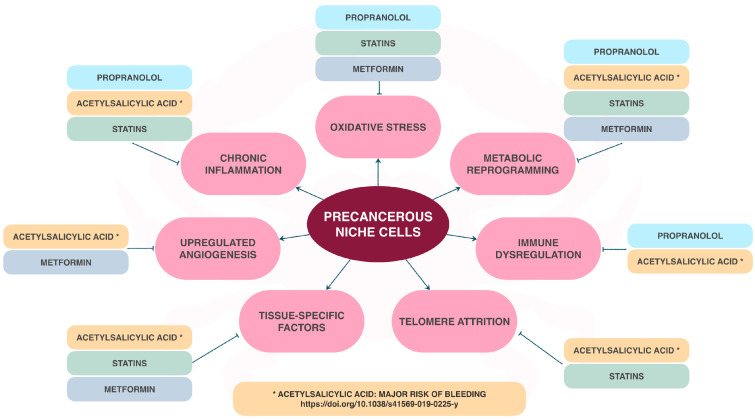
Proposed mechanisms of action for drugs targeting the precancerous niche [13,25,26].

Studies about chemoprophylaxis in LFS are limited. In 2016, an LFS mice model was treated with metformin, a derivative of the plant *Galega officinalis*, extending cancer-free survival compared to the control group [23]. In 2020, when LFS patients were administered metformin, researchers observed changes in mitochondrial function and reduced tumor cell proliferation. These effects were accompanied by decreased levels of insulin-like growth factor 1 (IGF-1) and insulin-like growth factor binding protein 3 (IGFBP3), which were associated with *mTP53* activity [24]. This study led to the formulation of the “Metformin in adults with Li–Fraumeni syndrome” (MILI) trial, currently in phase two, which is the only reference for LFS chemoprophylaxis [25]. Other therapies under investigation include statins, acetylsalicylic acid, and propranolol (Figure 1) [13,25].

### 1.7. The Impact of Natural Compounds on Cancer

The impact of natural compounds on cancer pathogenesis is not new. An antioxidant and prooxidant theory has been described, particularly in acute myeloid leukemia, where polyphenols coming from natural products exhibit protective activity on healthy cells while promoting apoptosis in leukemic cells [27,28]. Therefore, understanding the impact of natural products on neoplastic diseases is essential.

**Figure 2:**
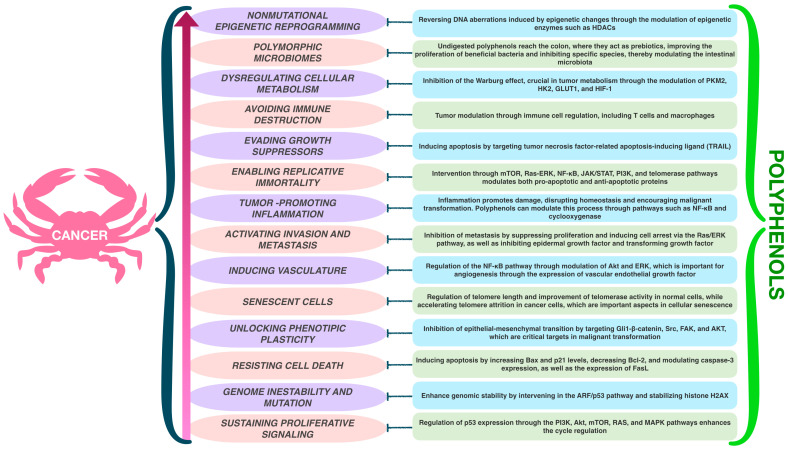
Mechanisms where polyphenols can act on the hallmarks of cancer [29,30,31,33,35,38,40,41,42,43,44,45,46,47,48,49,50].

One of the most notable characteristics of cancer is the diversity of factors that cause oncogenesis [29,30,31]. Therefore, considering its prevention requires targeting multiple pathways. The literature provides evidence that polyphenols, coming from natural compounds, actively influence this cancer pathogenesis (Figure 2). By referring to the cancer hallmarks proposed by Hanahan et al. [29,30,31], we observe that polyphenols target key aspects: they limit tumor cell proliferation [32], modify epigenetic reprogramming [33], modulate immune responses [34], modify microbiota [35], affect cellular senescence [36], induce apoptosis [37], regulate metabolism [38], and prevent genomic instability [39], which opens up the possibility of considering this as a chemoprophylactic measure.

### 1.8. The Role of Sechium Edule in Chronic Degenerative Diseases

In this context, studies have been conducted with the *Sechium edule* genus fruits, focusing on proliferative and metabolic diseases whose targets are closely associated with precancerous niches [51,52]. In vitro experiments have shown effects inducing apoptosis in pulmonary fibrosarcoma cells, cervical cancer [53], breast cancer [54], and leukemia cells with activity comparable to chemotherapy [27]. In vivo murine studies involving breast cancer demonstrated prolonged survival and reduced tumor size, similar to chemotherapy [55]. Metabolically, *Sechium edule* is being investigated as an adjunctive therapy in patients with metabolic syndrome, showing benefits across multiple biochemical and anthropometric parameters [51,52]. These activities are associated with a high content of polyphenols such as naringenin, rutin, and cucurbitacins B, D, E, and I [51,52,56,57]. However, these studies have been conducted in specific contexts without describing the potential impact of these compounds on chemoprophylaxis in hereditary mutations that predispose to cancer development like LFS.

### 1.9. Sechium H387 07 and Its Intervention in Precancerous Niche

The *Sechium* H387 07 hybrid was developed by the Mexican Interdisciplinary Research Group on *Sechium edule* (GISeM A.C), through the induced crossbreeding of *Sechium edule* (Jacq.) Sw groups, the varietal group *virens levis* and *amarus sylvestris* resulting in the hybrid H387 07 and registered in the National Inspection and Certification Service of Seeds (SNICs), under the Ministry of Agriculture, Livestock, Rural Development, Fisheries, and Food, Mexico, authenticated the fruits [58].

Belonging to the Cucurbitaceae family and the *Sechium* genus, it is a perennial, monoecious climbing plant with branched tendrils. The leaves are alternate, broadly ovate-orbicular. Male flowers are in axillary racemes with filaments united in a column, while female flowers are solitary or geminate. The fruit has a slightly obovate to predominantly pyriform shape. Its mesocarp is dark green with an intensely bitter taste and has minimal spongy material attached to the mesocarp. The seed size ranges from 0.7 to 6.3 cm, from 0.6 to 6.0 cm in width, and from 0.1 to 1.5 cm in depth. The *Sechium* hybrid fruits were collected by the GISeM at the germplasm bank of Cruo-Uach, Veracruz, Mexico [58].

**Figure 3:**
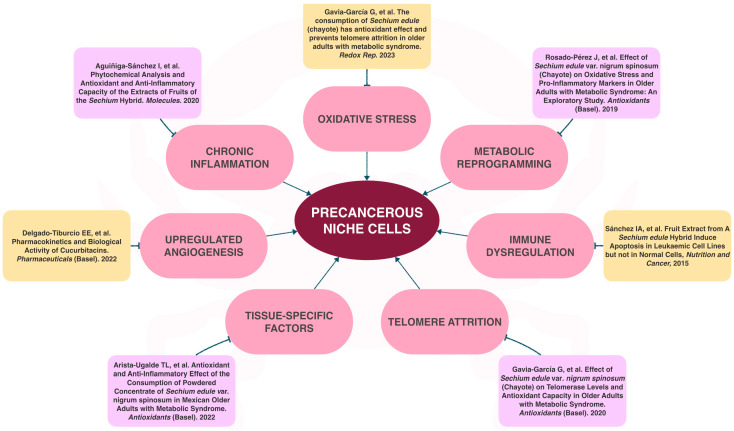
Polyphenols, derived from natural compounds found in *Sechium* capsules, may actively influence pathogenesis at action sites within the precancerous niche [13,27,39,52,56,57,61,62].

This hybrid has a significantly higher polyphenol content than its edible counterparts previously studied, as described by Aguiñiga and team. High-performance liquid chromatography revealed the hybrid’s content includes galangin at 21.94 mg/g, phloretin at 4.61 mg/g, naringenin at 3.3 mg/g, rutin at 1.27 mg/g, and myricetin at 0.889 mg/g, among others such as phenolic acids like gallic acid, chlorogenic acid, and syringic acid, in addition to cucurbitacins I, D, B, and E [55,57,59,60]. Currently, therapeutic options for LFS are limited, resulting in an ominous prognosis and mortality associated with malignancy. In this way, compounds that target precancerous niches while extending cancer-free survival are found to be essential. Previous evidence suggests that *Sechium* H387 07 extract may play a role by blocking critical sites within the precancerous niche (Figure 3).

This highlights the necessity to explore the potential impact of *Sechium* H387 07 extract to reduce the risk of developing new tumors in LFS. This study provides an extensive review of LFS, incorporating clinical, biochemical, and imaging data from over 11 years of *Sechium* H387 07 supplementation in an LFS patient with previous diagnoses and treatment of multiple malignancies. The research aims to offer a therapeutic alternative that prolongs cancer-free survival and enhances the quality of life for these patients.

## 2. Case Description

The patient is a 62-year-old female from Michoacan who works as an editor. In 2013, she came to this research unit at the age of 51; she presented with asthenia, adynamia, joint pain limiting passive mobility, painful adenopathy (six), and cutaneous neoformations on the facial region.

### 2.1. Literature Review

For this research, a search was conducted in multiple specialized databases related to LFS, *Sechium edule,* and the antiproliferative properties of plant compounds. These databases included PubMed/Medline, Scopus, DynaMed Plus, EMBASE, and ScienceDirect. A catalog of theses from the National Autonomous University of Mexico (UNAM) was also consulted. Search terms used included “Li–Fraumeni Syndrome”, “*Sechium edule*”, “*Sechium* H387 07”, “*TP53*”, “Tumoral microenvironment”, and “Flavonoids”. Language restrictions were applied to English and Spanish, and documents were selected prioritizing recent publications.

### 2.2. Family History

There is a history of cancer on her paternal side. Her father had renal cancer at age 63 without genetic counseling, and among her six siblings, two are deceased, the cause of death for one sibling remains undetermined, occurring in the first year of life. In contrast, the second sibling’s death was associated with unspecified central nervous system neoplasia at two years. Three siblings carry a likely pathogenic/pathogenic variant in *TP53* (p.Gly279Glu; ClinVar variation ID: 419454). Their cancer histories are as follows:

1. Debut at age 34 with breast cancer, thyroid cancer, radio-induced osteosarcoma, dealing currently with glioblastoma.
2. Debut at age 45 with lung cancer, currently at stage IV.
3. Debut at age 35 with breast cancer, thyroid cancer, and currently stage IV lung cancer.

### 2.3. Personal History

Regular Mexico-American diet, average sun exposure of one hour daily before age 35, passive smoking exposure. Menarche at 12 years, sexually active since 18 years, oral contraceptives from ages 18 to 30, five pregnancies, four abortions, and one live birth carrier of likely pathogenic/pathogenic *TP53* variant. Underwent total abdominal hysterectomy with bilateral salpingo-oophorectomy in 2004, last cervical cytology and colposcopy in 2015, showed no abnormalities.

Diagnosed with breast cancer at 44 years, ECIIB stage (T2N3M0), 17/28 positive lymph nodes in the left half of the body. Histopathological diagnosis revealed grade III infiltrating ductal carcinoma without specific patterns, triple-negative breast cancer with p53 4+ mutation. She underwent radical mastectomy (June 2006), received six cycles of adjuvant chemotherapy, and underwent radiotherapy with 50 Gy in 25 fractions. Definitive LFS diagnosis by polymerase chain reaction (PCR) (July 2012) with a likely pathogenic/pathogenic variant at p.G279E (replacing glycine with glutamate at codon 279; ClinVar variation ID: 419454). Underwent risk-reducing total right mastectomy (October 2014). Developed pleomorphic fibrous histiocytoma in the left scapular region at age 47, requiring surgical resection (January 2009) and developed radio-induced pleomorphic sarcoma in left costal region at age 52 requiring bloc resection of left rib cage with mesh placement (January 2015).

After discussing the antiproliferative effects of *Sechium* H387 07’s extract in vitro and in vivo and considering the absence of therapeutic alternatives, a joint decision was made to initiate oral supplementation.

### 2.4. First in Human (FIH) Calculation

The dosage of *Sechium* H387 07 extract was calculated based on toxicology studies [63], which recommend starting with a dose equivalent to 1/6 of the non-severe toxic dose (25 mg/kg), a dose documented in a mice model [64].

$$\mathrm{FIH}=\frac{Estimated\;weight\;in\;grams\;\left(75,000\right)\times Non\text{-}severe\;toxic\;dose\;\left(25\right)\;÷\;Conversion\;to\;kg\;(1000)}{6}=312.5\;\mathrm{mg}\;*$$

* Rounded to 300 mg/day.

### 2.5. Capsule Elaboration from the Extract of Sechium H387 07

Fruits of *Sechium* H387 07 obtained from the National *Sechium* Germplasm Bank in Mexico were used [65]. The fruits were harvested at horticultural maturity, washed, dried, and cut into flakes. Subsequently, they were placed in an air-circulating oven at 40 °C for complete dehydration, lasting approximately 48 h. The dried material was ground to a particle size of 2 mm and sieved through a No. 4 mesh. Finally, the biological material was encapsulated in gelatin capsules containing 150 mg and stored in labeled containers holding approximately 90 capsules. These containers were kept protected from light in cool, dry storage conditions at room temperature in Mexico City. The capsules were administered to our patient within the first month of production, with doses for three months, ensuring similar storage conditions were maintained at the patient’s residence for continued supplementation.

### 2.6. Implemented Intervention

Before informed consent and based on the calculated FIH dose (300 mg/day), 150 mg of *Sechium* H387 07 capsules was administered every twelve hours continuously over 11 years, starting in April 2013 and continuing to the present.

### 2.7. Laboratory, Histopathology, and Imaging Studies

Laboratory samples, blood counts, blood chemistry, and histopathology analyses were conducted at various public and private institutions. The processing of samples adhered to Mexican regulations. Genomic testing was performed by Ambry Genetics using PCR and multiplex ligation-dependent probe amplification techniques targeting the *TP53* gene.

### 2.8. Full Body PET/CT

A scan was conducted at PET/CT unit UNAM using a Siemens biograph vision 600 scanner on 8 May 2024. F-18 Fluorodeoxyglucose (F-18 FDG) was administered with an injection starting at 16:36, at 192.40 MBq. Considering a weight of 76 kg, a height of 1.59 m, and an effective dose of 3.66 mSv, scanning occurred from 17:38 to 17:43, with a total duration of 970 s.

## 3. Discussion

A continuous follow-up was conducted from the start of treatment in April 2013, with clinical, laboratory, and imaging records to assess the impact of the *Sechium* H387 07 compound on the patient.

### 3.1. Administration and Adverse Effects

After administration of *Sechium* H387 07 capsules, the patient experienced intestinal rumbling and decreased consistency of stools without altering frequency (1 to 2 per day). These effects did not meet diarrhea criteria [66] and have remained unchanged up to the present moment, with a maintained dosage of 300 mg/day.

### 3.2. Malignancy Follow-Up

**Figure 4:**
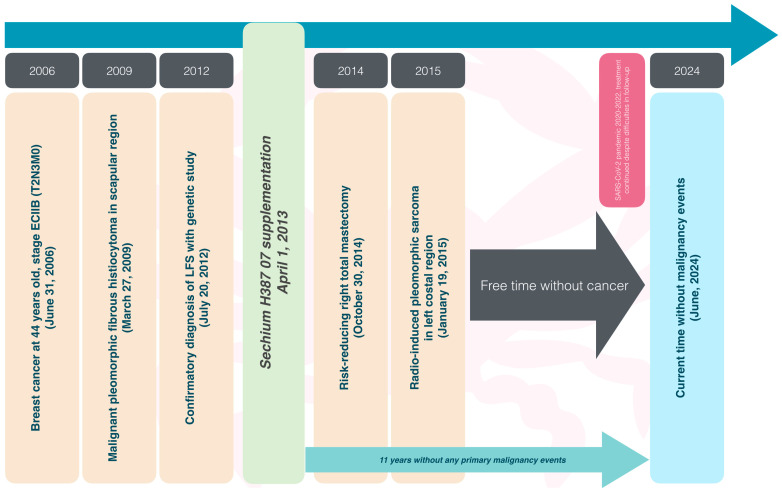
The chronological sequence of malignant events and the supplementation of *Sechium* H387 07 capsules throughout Li–Fraumeni syndrome.

A focused follow-up was conducted regarding malignancies. The last primary malignancy episode occurred in 2009 with a pleomorphic fibrous histiocytoma. In 2014, radio-induced pleomorphic sarcoma secondary to breast cancer radiotherapy (2006) required surgical resection of the left rib cage with mesh placement. After this, our patient has been cancer-free for ten years since the surgical resolution of the sarcoma found a strong correlation with the intake of *Sechium* H387 07 capsules (Figure 4).

### 3.3. Clinical Follow-Up

**Figure 5:**
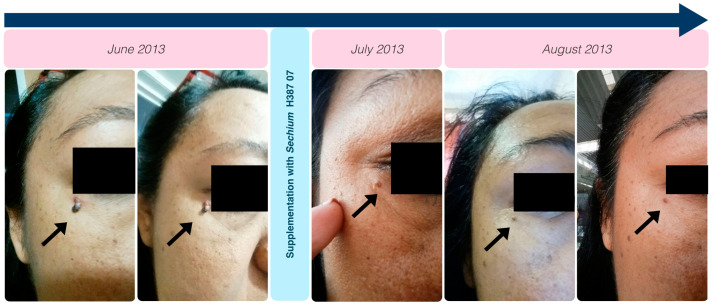
Chronological development of neoplastic formations preceding supplementation, with the changes observed after supplementation. The black arrow indicates cutaneous neoformation and its reversion within the first months of supplementation.

After starting the supplementation of *Sechium* H387 07 capsules, cutaneous neoformations regressed (Figure 5). Additionally, the patient experienced overall improvement, with a resolution of asthenia, adynamia, and arthralgias. Improved passive mobility allowed her to resume her activities over the years. There are no clinical signs of involvement in any evaluated systems except for minor movement-related discomfort due to the rib cage’s resection.

### 3.4. Laboratory Follow-Up

Laboratory studies have consistently shown stable parameters since 2006, even before supplementation. Hematological malignancies were followed up based on values within normal ranges for the red blood cell index, leukocyte, and platelet count. Last metabolic parameters indicate an elevated serum glucose level due to non-fasting conditions during sample collection. To verify this, an HbA1c test was conducted in May 2024, obtaining 5.5%; this value does not meet the threshold for diabetes according to ADA [67]. Additionally, mixed dyslipidemia has been observed, but values remained consistent before treatment, ruling out a causal association.

Liver function tests showed no differences compared to pre-treatment levels. The calculated FIB-4 index of 0.91 corresponds to Ishak stage 0–1 with minimal fibrosis. Renal parameters have remained stable since initial assessments, without proteinuria and a glomerular filtration rate of 83 mL/min/1.73 m^2^ in 2024 (CKD-EPI), classified as stage 2 according to KDIGO criteria [68]. All these values in normal ranges (Table 1) are essential to discard treatment-related toxicity.

### 3.5. PET/CT Follow-Up

**Figure 6:**
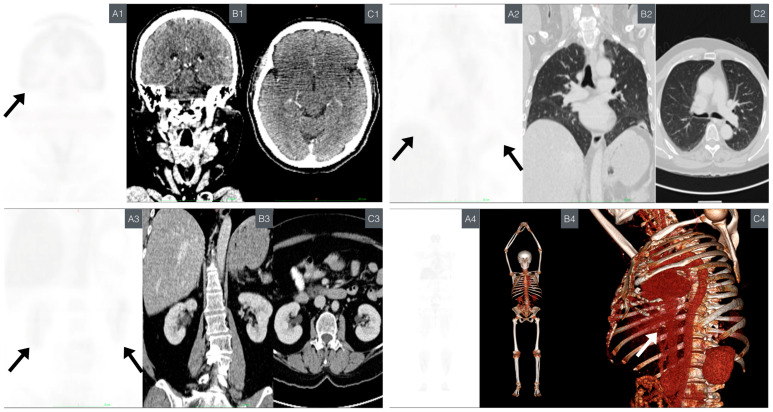
Comparative PET/CT imaging. This highlights the primary organs commonly affected by cancer in patients with LFS. The black arrow on the PET scan indicates the principal organs affected by LFS. PET: displayed on the left (**A**), showing SUV ranging from 0.0 to 1.0 g/mL. CT: presented in the middle for anatomical reference (**B**). Axial slide CT (**C**). (**A1**–**C1**) Comparative neuroimaging from SNC. (**A1**) PET: coronal brain section. (**B1**) CT: coronal brain section corresponding to the location in image “A”. (**C1**) CT: axial brain section. (**A2**–**C2**) Comparative imaging from the chest. (**A2**) PET: coronal lung section. (**B2**) CT: coronal lung section corresponding to the location in image “**A**”. (**C2**) CT: axial lung section (**A3**–**C3**) Comparative imaging from the abdomen. (**A3**) PET: coronal kidney section. (**B3**) CT: coronal kidney section corresponding to the location in the image “**A**”. (**C3**) CT: axial kidney section. (**A4**–**C4**) Whole-body comparative imaging. (**A4**) PET: coronal section of the body. (**B4**) CT: three-dimensional modeling highlighting bone structures. (**C4**) CT: three-dimensional thoracic modeling focused on the lateral thorax, visualizing the surgical site (white arrow). Abbreviations: central nervous system (CNS), computed tomography (CT), Li–Fraumeni syndrome (LFS), positron emission tomography (PET), standardized uptake value (SUV).

Due to the unavailability of whole-body magnetic resonance imaging (MRI), the preferred imaging modality, PET/CT, was chosen as a screening study for cancer. This decision was made considering its high sensitivity and specificity. Although this imaging modality is still under study due to the use of radiation, it is recommended in LFS screening guidelines because of its significant role in offering benefits in terms of spatial resolution, a major limitation of MRI [69,70,71,72]. Emphasis was placed on areas with higher cancer incidence in LFS patients and particularly those present in direct relatives. These areas were evaluated within standardized uptake value (SUV) ranges of 0.0–1.0 and selected as representative images (Figure 6). More detailed photos, including fusion mode, are provided in Supplementary Figures S1–S4.

The most significant findings include the following:

- Left rib region increased inflammatory metabolism: metabolic activity was observed in the corresponding area to the resection site with mesh placement. The SUVmax value was 2.8, falling within the expected and favorable prognostic range (<10.2 SUVmax) [73].
- Lung base subsegmental atelectasis: subsegmental atelectasis was noted in lung bases, along with a nodule in the lower lingula. The SUVmax for the nodule was 0.8, which is below the predictive malignancy cutoff value (SUVmax > 2.5) [74].

No evidence of metabolic or morphological abnormalities was found in other organs or structures.

### 3.6. Analysis of the Intervention of Polyphenols Present in Sechium on the Precancerous Niche

Patients with LFS develop cancer at some point in their lives, compromising their quality of life and increasing morbimortality. After cancer establishment, treatment options such as radiotherapy or chemotherapy are limited due to the risk of inducing secondary neoplasms [7,12]. Additionally, the natural history of disease shows a high risk of developing a second primary cancer within ten years after the first diagnosis, further overshadowing the prognosis [9], which requires finding alternatives for prevention. A tumor microenvironment that promotes oncogenesis has been described in LFS patients. Research has explored metformin use (Figure 1), a phytochemical derivative, as a prophylactic measure due to its ability to target multiple aspects of the tumor microenvironment [13,25]. *Sechium* H387 07 contains phytochemicals that exert this activity due to its content of polyphenols with demonstrated antineoplastic, antioxidant, anti-inflammatory, and hypoglycemic properties in preclinical and clinical studies [39,51,52,53,55,57,64].

After presenting two primary tumors and one secondary tumor, we are pleased to report that our patient with LFS has remained free of new primary tumors or recurrence for 11 years with *Sechium* H387 07 capsule supplementation. This outcome contrasts with siblings who have shown a high disease penetrance and experienced worse outcomes without consuming this supplement. High-sensitivity imaging studies have revealed no signs of malignancies, including hematological malignancies, through laboratory assessments, including complete blood counts, and have not indicated any hepatic or renal compromise associated with the treatment on biochemical tests; also, the patient has reported improved quality of life and resolution of symptoms.

**Figure 7:**
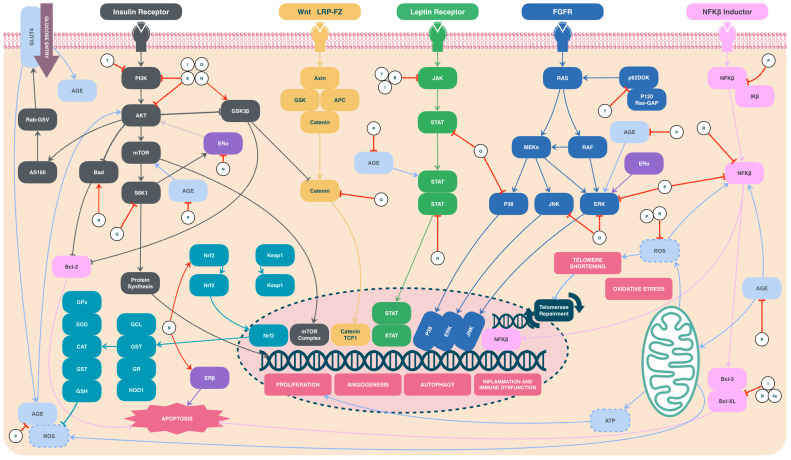
Action sites of *Sechium*’s polyphenols. Abbreviations: AGE—advanced glycation end products; B—cucurbitacin B; E—cucurbitacin E; I—cucurbitacin I; IIa—cucurbitacin IIa; HG—hyperglycemia; N—naringenin; P—polyphenols; Q—quercetin; R—rutin; T—protein tyrosine phosphatase 1B (PTP1B) [39,51,52,56,57,64,79,80,81,83,86,87,89,92,93,94,95,96,97,98,99,100,101,102].

This evidence underscores the chemoprophylactic potential effect of *Sechium* H387 07 on the tumor microenvironment through its phytochemicals. These compounds impact various precancerous niches associated with *mTP53* mutations, including oxidative stress, metabolic reprogramming, telomere shortening, and apoptosis. These phytochemicals in *Sechium* elucidate its chemo-prophylactic properties, as demonstrated in our findings (Figure 7).

- Oxidative Stress/Immune Dysregulation/Metabolic Reprogramming/Angiogenesis: these pathways are closely associated and are particularly affected in family members carrying *mTP53* mutations. Individuals with these pathogenic variants exhibit increased oxidative phosphorylation and oxidative stress compared to healthy relatives. This prooxidant state leads to inflammation and DNA damage, predisposing to cancer development [13,75]. Recent studies have demonstrated that inhibiting oxidative stress can prevent oncogenesis [76]. Additionally, p53 influences AKT/mTOR pathways critical for regulating proliferation, survival, glucose metabolism, and amino acid use, and where, in the presence of *mTP53*, anaerobic glycolysis (Warburg effect) is stimulated, favoring the tumor microenvironment [77,78]. Persistent inflammatory conditions also stimulate vascular growth factor synthesis. This perpetuation occurs in cases where *TP53* is absent because it normally modulates antiangiogenic factors via proteins like thrombospondin-1 (TSP-1) [79]. *TP53* regulates toll-like receptors (TLR), which are essential in the tumor microenvironment. In the presence of *mTP53*, recognition of premalignant cells is impaired, facilitating malignant transformation, inflammation, and aberrant cytokine production, perpetuating the proinflammatory state [80,81]. Some interventions through antioxidant and anti-inflammatory factors are described below:
  ○ Instead, it is known that polyphenols counteract these activities. Thus, polyphenols can mediate proliferation, cell cycle, and arachidonic acid pathways by regulating transcription factors such as PI3K, STAT, and MAPK. They can also inhibit aberrant TLR and have a significant metabolic impact by increasing high-density lipoprotein (HDL) and reducing low-density lipoprotein (LDL), thus preventing the production of oxidized LDL (an essential source of vascular comorbidities) [51,52,82,83].
  ○ On the other hand, naringenin or rutin donates hydrogen atoms to OH groups, stabilizing molecules and limiting damage to free radicals. Consequently, they can inhibit the release of nuclear factor kappa B (NF-κB), subsequently reducing proinflammatory cytokines [51,52,57,83,84]. Additionally, they play an essential role in Keap-Nrf2 overexpression, inducing antioxidant production rather than inhibiting oxidant enzymes [51,52,57,64,85].
  ○ In glycolysis disorders, the hyperglycemic states promote nucleotide, lipid, and amino acid synthesis, which are necessary for the proliferation, invasion, and migration of malignant cells. *Sechium* H387 07 extract, with hypoglycemic and antioxidant effects, improves disease control in patients with metabolic syndrome [51,52,57,64]. This effect is attributed to PI3K pathway inhibition (which modulates glucose transporters) combined with oxidative stress reduction, potentially reversing insulin resistance and mitigating metabolic disorder effects [86].
  ○ Regarding the role of tyrosines and protein tyrosine phosphatase 1B (PTP1B) in breast cancer development, tyrosines play a role through PTP1B in estrogenic and proliferation pathways. Aberrant PTP1B activity, present in breast cancer, can lead to oncogenesis [87,88]. This is particularly relevant in patients with LFS due to the high incidence of breast cancer. Preclinical studies have shown that silencing aberrant PTP1B activity inhibits proliferation, and *Sechium edule* has been documented as a potential inhibitor of this tyrosine [89].
  ○ Telomere shortening is a natural process associated with aging, marking the finite replicative capacity of cells. However, excessive telomere shortening is pathological and linked to oxidative stress, which can predispose to mutagenesis [39,90]. Early-onset cancer incidence in LFS has been associated with excessive telomere shortening due to MDM2 signaling disturbances that promote genomic instability and increase cancer susceptibility [91,92], highlighting it as an important therapeutic target. Applying *Sechium edule* in patients with metabolic syndrome has shown telomere length maintenance without altering telomerase levels, an effect closely associated with its antioxidant capacity [39,90]. Additionally, polyphenols have the potential to overexpress and stabilize p53, an opportunity to reactivate aberrant p53 and regulate transcription factors such as MDMX and MDM2 through phosphorylation and acetylation, favoring telomere length [32].
  ○ In apoptosis, *TP53* plays a crucial role in cell cycle regulation by controlling transcription factors associated with DNA repair, such as GADD45 and PCNA. It acts as a direct apoptosis regulator, inhibiting survival genes like anti-apoptotic *BCL2* and upregulating pro-apoptotic genes like *BAX*. However, this capacity is compromised in *mTP53* presence, promoting tumor development. Stimulating pro-apoptotic pathways becomes necessary [93]. Naringenin, also present in *Sechium* H387 07 extract, inhibits Prdx-1, an important ASK1 inhibitor in programmed cell death, and upregulates both extrinsic (TNFRST10D/CRADD/CASP-2) and intrinsic (PTEN/BBC3/APAF-1/CASP-9) apoptosis pathways. Naringenin also promotes overexpression of estrogen receptors (ERβ), which regulate apoptosis via p38/MAPK, while inhibiting ERα, responsible for proliferation [37,94,95].
  ○ Overexpression of PI3K/AKT pathways is associated with poor prognosis in some cancers [96,97]. Quercetin, rutin, and cucurbitacin I inhibit this pathway, leading to cell cycle arrest and caspase production [98]. They also induce apoptosis due to PI3K/AKT’s role in regulating Bcl-2 and Bax proteins. Quercetin additionally inhibits critical Wnt/β-catenin pathways involved in proliferation, stimulates ferroptosis via lipid peroxidation [99], and induces G2/M cell cycle arrest through p-STAT modulation and favoring caspase pathways via LC3/ERK/Caspase-3 [100].
  ○ The FAK/AKT/GSK3β pathway is regulated by p53. In the presence of *mTP53*, it is overexpressed, promoting proliferation, metastasis, and angiogenesis [26,101]. Cucurbitacin E has been shown to analogously inhibit phosphorylation induced by these pathway proteins [102].

Thus, the involvement of polyphenols from *Sechium* H387 07 compound in targeting the tumor microenvironment is extensive, highlighting its appropriate application to our patient, who has shown favorable progress compared to direct relatives. Future research should explore the impact of this supplementation on a more significant number of patients and consider other conditions with predisposition to cancer. Furthermore, evaluating its effects once cancer has been established is crucial. This study shows how the polyphenols can simultaneously target areas also impacted by chemotherapy, thus opening a new field of study with potential applications.

Finally, given the high concentration of polyphenols, it will be essential to investigate their interactions at the physiological level and with drugs in a population with comorbidities where polypharmacy is common. Recent descriptions have highlighted the role of these compounds in physiological processes, including their interaction with CYP450 and certain drugs [103,104,105]. Even when no adverse effects were found in our patient over the past eleven years, further research is necessary to identify possible pharmacological synergies and avoid antagonisms. Continued treatment through case series will be essential to recommend its use within the clinical context.

## 4. Conclusions

This study describes the follow-up of a patient with LFS who has experienced eleven years of primary tumor remission associated with *Sechium* H387 07 supplementation. This represents the first evidence of the antineoplastic potential of *Sechium* in LFS patients. The literature review indicates that compounds from *Sechium* H387 07 have a corrective effect on a precancerous niche, which explains the extension of cancer-free survival and improved life quality through a simple and well-tolerated therapeutic approach. This is in contrast to direct relatives who have experienced a more deleterious disease course.

Based on these findings and considering its advantages, such as greater accessibility, a simple therapeutic approach, better tolerability, and improved effectiveness compared to other alternatives, *Sechium* H387 07 demonstrates a powerful chemoprophylactic effect by targeting multiple key sites simultaneously. Therefore, we aim to promote further investigation and clinical application of *Sechium* H387 07 and polyphenols in more prevalent proliferative conditions. This will be pursued through clinical trials involving a more significant number of patients, aiming to expand the arsenal of therapeutic options against cancer and improving the quality of life for our patients.

## Figures and Tables

**Table 1:** Laboratory test’s evolution.

Laboratories	June 2006	January 2007	November 2010	February 2011	October 2012	May 2013	November 2014	September 2015	February 2024
Hemoglobin (g/dL)	14.7	13.3	14.1	15.1	14.7	15	14.6	14.9	16.2
Hematocrit (%)	44.1	40.2	44	46.1	45.2	45.8	42.9	45.6	51.2
Erythrocytes (mm^3^)	5,250,000	4,800,000	5,150,000	5,180,000	5,220,000	5,320,000	5,090,000	5,310,000	6,090,000
Leukocytes (mm^3^)	10,000	4060	7100	4800	8100	6500	8000	7100	6060
Neutrophils (mm^3^)	6300	2400	4260	2980	4430	4000	5410	4300	3490
Lymphocytes (mm^3^)	3400	1200	2556	1060	2580	1810	1910	1980	1880
Monocytes (mm^3^)	200	200	142	610	670	420	400	460	316
Eosinophils (mm^3^)	100	200	142	130	400	210	220	330	190
Basophils (mm^3^)	0	0	0	10	70	50	60	30	140
Platelets (mm^3^)	366,000	260,000	370,000	251,000	289,000	289,000	357,000	295,000	334,000
Glucose (g/dL)	73	98	83	64	92	83	81	87	119
Urea (g/dL)	20	24	39	-	25	15.6	-	37	32.1
Uric acid (g/dL)	5	-	5.2	6.7	6	5.9	6.6	5.5	6.8
BUN (g/dL)	9.35	11.1	18.2	10.0	-	-	16.8	17.3	15
Creatinine (g/dL)	0.9	0.91	0.7	0.7	0.81	0.7	0.7	0.7	0.8
Cholesterol (g/dL)	234	222	309	218	301	321	282	285	304
Triglycerides (g/dL)	133	337	133	88	204	202	191	229	245
Direct bilirubin (g/dL)	0.1	0.11	-	0.2	0.16	0.12	0.09	0.12	0.16
Indirect bilirubin (g/dL)	0.6	0.32	-	0.5	0	1	0.61	0.7	0.87
AST/TGO (U/L)	22	22	-	30	24	24	20	21	29
ALT/TGP (U/L)	26	42	-	47	48	39	47	32	35
ALP (U/L)	70	105	-	111	116	116	115	114	-
LDH (U/L)	255	257	-	454	294	213	151	160	210

Chronology of blood tests and *Sechium* H387 07 capsule supplementation.

## Data Availability

The original contributions presented in this study are included in the article/Supplementary Materials; further inquiries can be directed to the corresponding author.

## Supplementary Materials

The following supporting information can be downloaded at: https://www.mdpi.com/article/10.3390/ijms252111477/s1.

## Abbreviations

Central nervous system (CNS), Computed tomography (CT), First in human (FIH), Li–Fraumeni syndrome (LFS), High-density lipoprotein (HDL), Mexican Interdisciplinary Research Group on *Sechium edule* (GISeM), Low-density lipoprotein (LDL), Magnetic resonance imaging (MRI), Mutant tumor protein 53 (*mTP53*), National Autonomous University of Mexico (UNAM), Nuclear factor kappa B (NF-κB), Polymerase chain reaction (PCR), Positron emission tomography/computed tomography (PET/CT), Protein tyrosine phosphatase 1B (PTP1B), Standardized uptake value (SUV), Toll-like receptors (TLR), Tumor protein 53 (*TP53*).
