# Supplementary figures and images for: Li–Fraumeni Syndrome: Narrative Review Through a Case Report with Ten Years of Primary Tumor Remission Associated with Sechium H387 07 Supplementation
Source: Int J Mol Sci. 2024 Oct 25;25(21):11477. doi: 10.3390/ijms252111477 (PMC11546847; doi:10.3390/ijms252111477)

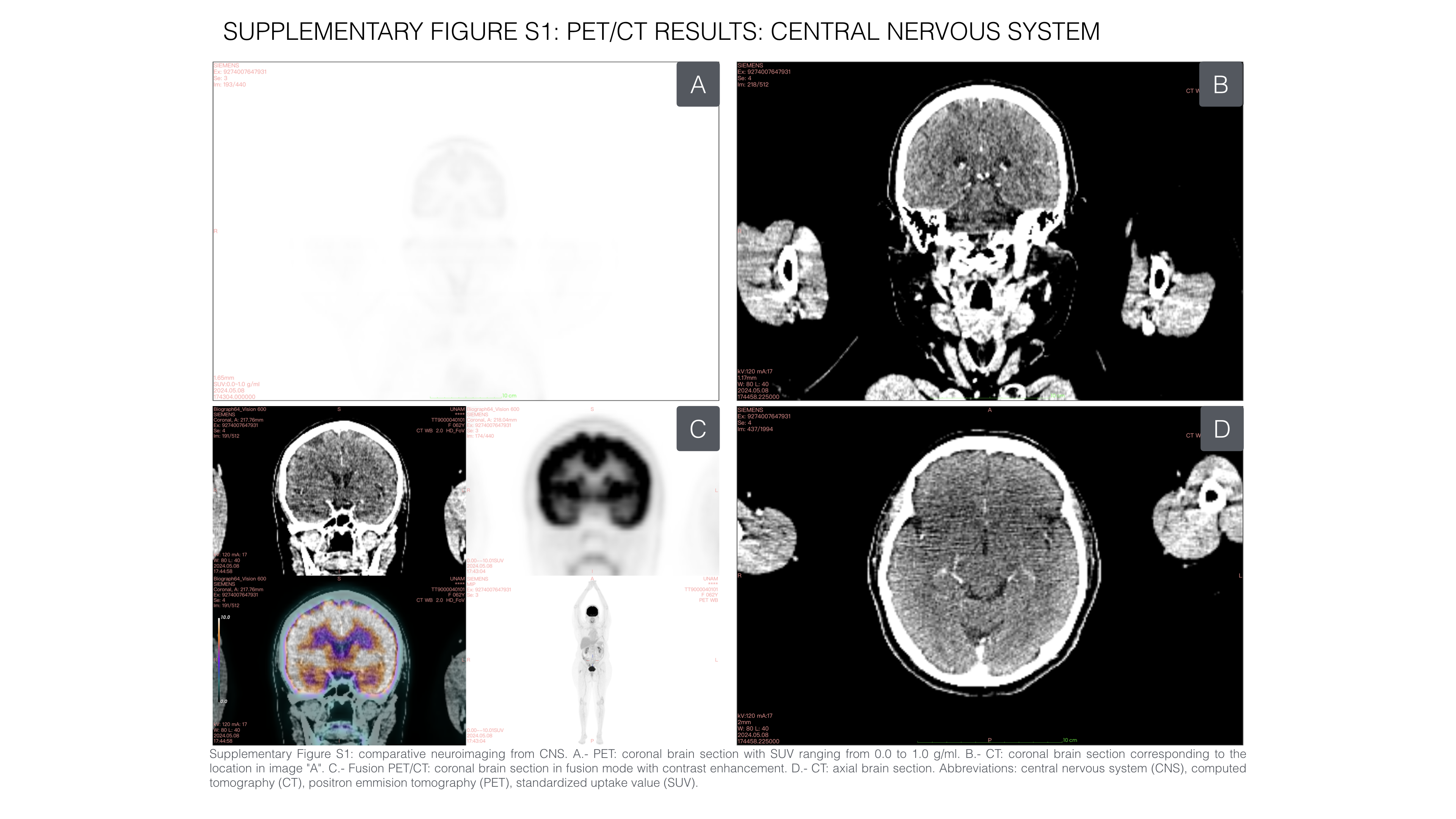

Supplement: Supplementary file 1 [file ijms-25-11477-s001.zip › Supplementary Figure S1.tiff]

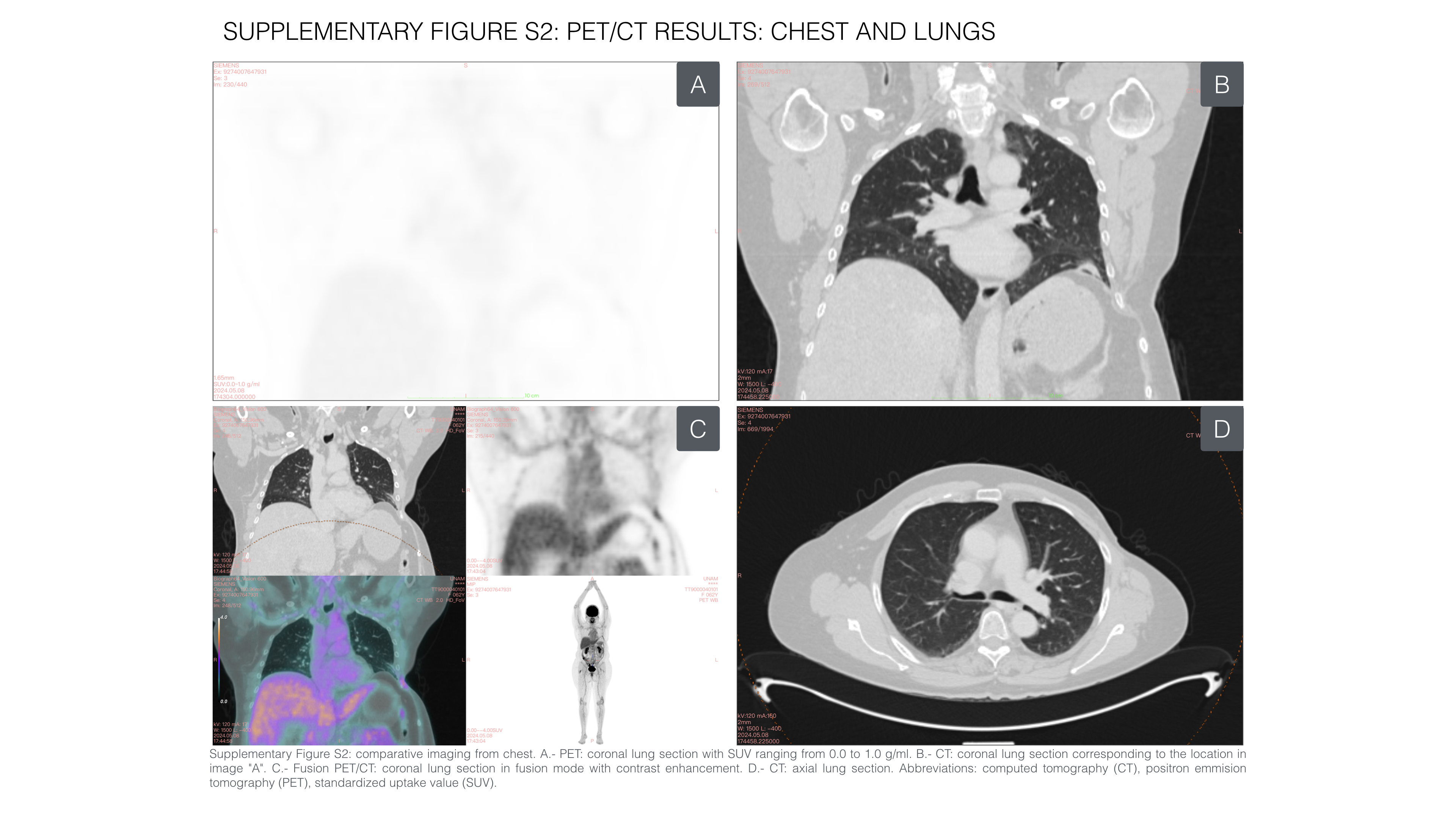

Supplement: Supplementary file 1 [file ijms-25-11477-s001.zip › Supplementary Figure S2.tiff]

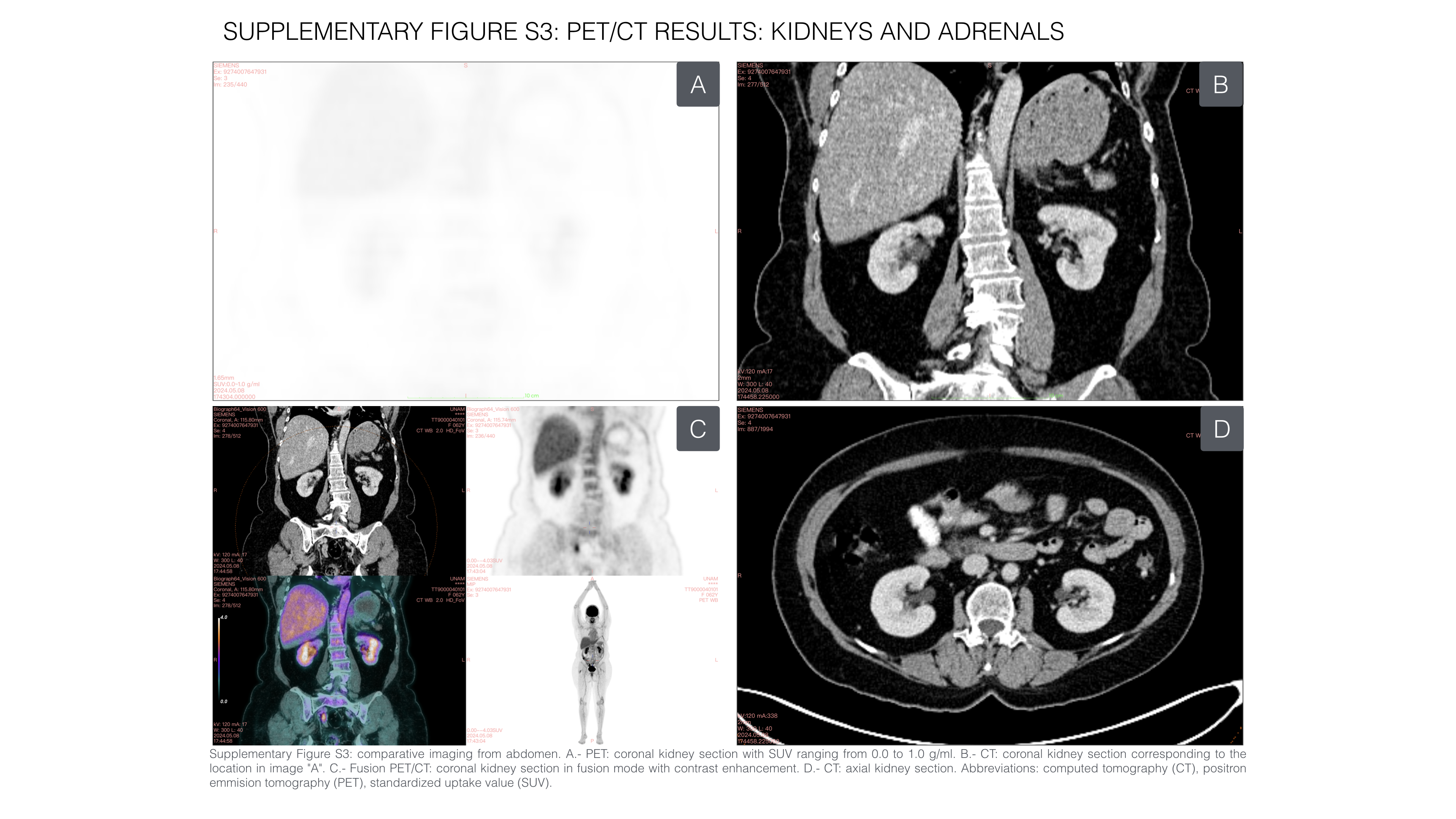

Supplement: Supplementary file 1 [file ijms-25-11477-s001.zip › Supplementary Figure S3.tiff]

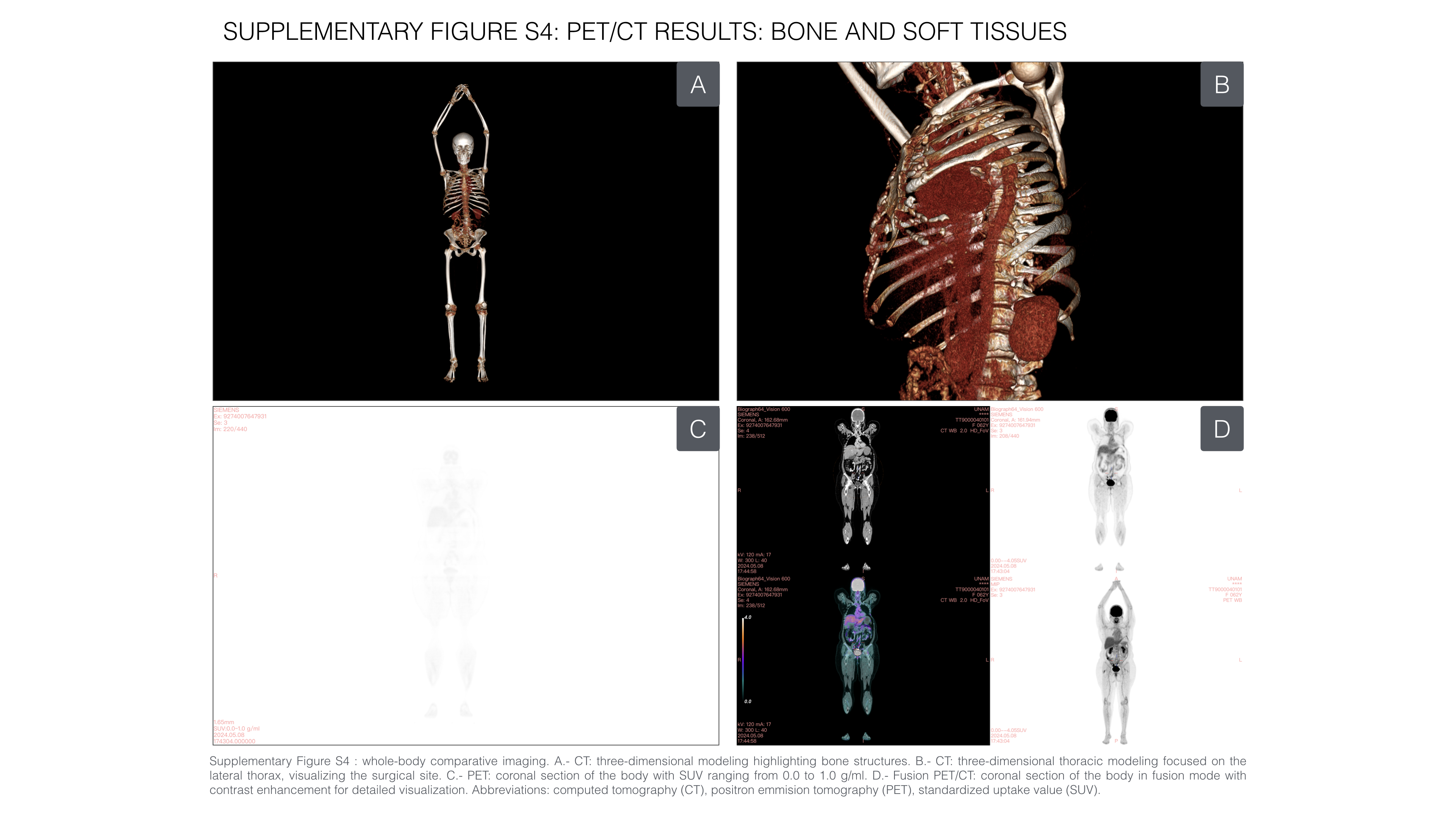

Supplement: Supplementary file 1 [file ijms-25-11477-s001.zip › Supplementary Figure S4.tiff]
